# Effects of COVID-19 Lockdown on Weight, Body Composition, and Behavior of Children, Adolescents, and Young Adults with Prader–Willi Syndrome

**DOI:** 10.3390/jcm10204746

**Published:** 2021-10-16

**Authors:** Andrea Karoline Mohr, Constanze Laemmer, Sandra Schulte, Bettina Gohlke

**Affiliations:** 1 Department of Pediatric Endocrinology and Diabetology, Children’s Hospital, University of Bonn, Venusberg-Campus, 53127 Bonn, Germany; sandra.schulte@ukbonn.de (S.S.); bettina.gohlke@ukbonn.de (B.G.); 2 Pediatric Endocrinology and Diabetology, St. Bernward Hospital, Treibestraße 9, 31134 Hildesheim, Germany; dr.c.laemmer@bernward-khs.de

**Keywords:** Prader–Willi syndrome, genetic obesity, COVID-19, physical activity, eating behavior, growth hormone

## Abstract

To reduce transmission of the coronavirus disease 2019 (COVID-19), many countries implemented lockdowns, causing the closure of childcare services. This study was designed to evaluate the impact of the COVID-19 lockdown in March–April 2020 on children, adolescents, and young adults with Prader–Willi syndrome (PWS) living in Germany. We recruited 180 participants with a genetically confirmed PWS. All families completed a questionnaire, and participants underwent a post-lockdown assessment; the last examination before the lockdown was determined as the pre-lockdown assessment. We used bivariate analyses to compare pre- and post-lockdown outcomes. Weight standard deviation scores (SDS_PWS_) and body mass index (BMI)-SDS_PWS_ remained stable or even decreased in some age groups. A statistically significant gain in lean body mass (LBM) was found in all groups <18 years of age. We observed an increase in IGF-I and IGFBP-3 concentrations without a significant change in growth hormone (GH) dosage. Most families (95.4%) reported set mealtimes and implementation of structured activities (72.2%) during the lockdown period. We therefore suggest that the favorable development of weight/BMI and LBM was caused by an interplay of a suspected enhanced GH administration and continuous parental commitment. However, more intense behavioral problems were observed in 45.7%, which persisted post-lockdown in 33.7%.

## 1. Introduction

Prader–Willi syndrome (PWS) is a complex multisystem disorder caused by the absence of the expression of paternally inherited imprinting genes on chromosome 15q11-q13 caused by a paternal de novo deletion, maternal uniparental disomy (UPD), or an imprinting center defect (ID) [1,2]. Cardinal features of the syndrome include neonatal hypotonia with feeding difficulties and failure to thrive, followed by hyperphagia and food-seeking behavior with onset in early childhood [1,3]. Individuals with PWS show an altered body composition, with increased adiposity and a decreased lean body mass (LBM) as a manifestation of growth hormone (GH) deficiency [4,5,6,7]. The reduction in muscle mass causes a reduced resting energy expenditure and avoidance of physical exercise. Together with increased energy intake due to hyperphagia, individuals with PWS show a high prevalence of severe obesity [8,9]. Apart from GH deficiency, common endocrinopathies in individuals with PWS include hypogonadism and hypothyroidism [6,7]. Other characteristic features include developmental delay and behavioral problems [2]. First-line management of PWS includes a low-calorie diet and physical exercise in conjunction with GH therapy, as GH improves body composition and, thus, metabolism [10,11]. Through strict management requiring multidisciplinary care, a normal body mass index (BMI) can be achieved [3,6]. 

At the beginning of 2020, there was a rapid global expansion of the coronavirus disease 2019 (COVID-19); on 11 March 2020, the World Health Organization (WHO) declared a worldwide pandemic [12]. To reduce transmission occurring through respiratory droplets, many countries imposed regulations of social distancing and lockdowns [13]. Children with PWS, who commonly attend special-need childcare services, had to be cared for at home, and therapy sessions were canceled. Therefore, families suddenly had to manage the care for their child with PWS without the support of a multidisciplinary team. This study was designed to assess the impact of the lockdown on children, adolescents, and young adults with PWS in Germany, focusing on changes in anthropometric parameters and body composition, as well as metabolic factors and behavior. 

## 2. Materials and Methods

### 2.1. Participants

The participants of this retrospective, longitudinal study were recruited at a single PWS center in Germany. Patients with a genetically confirmed PWS who visited the clinic between 4 May 2020 and 11 September 2020 were asked to participate. The study was approved by the Ethics Committee of the University of Bonn, Germany. All individuals underwent an examination, which was used as the follow-up assessment post-lockdown. The last assessment before the lockdown was determined as the pre-lockdown examination. Of 231 individuals with PWS visiting the clinic in 2020, we collected data of 186; 6 participants were excluded due to missing pre-lockdown examinations, resulting in a final sample of 180 patients (95 female). In the final sample, the genetic subtype had not been determined in 41%; 28% had a UPD, 22% a deletion, 3% an ID, and 6% a non-deletion subtype, which had not been differentiated into UPD or ID. Thirteen children (7%) had an additional disease (prematurity < 30 gestational weeks, type I diabetes mellitus, medically treated seizures, Triple X syndrome, spinal malformation). Twenty individuals (11.1%) took Levothyroxine for the treatment of hypothyroidism during the lockdown. In the group aged 11–17 years, five boys (20%) and two girls (10%) received sex steroids; in the young adults, twelve women (100%) and nine men (60%) took hormone replacement therapy. The participants were primarily Caucasian; ethnic minorities were not represented. Median interval between pre-lockdown and follow-up was 6.25 months in children and nine months in adults. Table 1 shows patients’ characteristics.

### 2.2. Anthropometric Parameters and Body Composition

Height and weight were measured at both assessments. Height was assessed using a rigid stadiometer, and weight was measured to the nearest 0.1 kg on a mechanical scale. BMI was calculated as weight/height^2^. We calculated standard deviation scores (SDS) according to age and sex for height, weight, and BMI using reference values for children with PWS (SDS_PWS_) [14]. Body composition was determined at both time points using bioelectrical impedance analysis (BIA) (Nutribox, Data Input). BIA is a non-invasive method, which relies on the different electrical properties of biological tissues. The assessment was performed after an overnight fast in a prone position. Patient information (sex, age, height, and weight) was entered, and patients were asked to lie still for five minutes to ensure an even blood distribution. During the analysis, two electrodes generate a weak electrical current inside the body. Total impedance is proportional to the conductor’s length and inversely proportional to its cross-sectional area. Muscle mass, containing water and dissolved electrolytes, conducts the electrical current, whereas bone and adipose tissue act as insulators. The software calculates LBM (kg), fat mass (kg and percent), and total body water (L) [15,16]. Due to a lack of reference values for children aged 0–3 years, children below the age of three were excluded from this analysis. 

### 2.3. Hormonal Parameters, Lipoproteins and Carbohydrate Metabolism

IGF-I concentrations were measured by an immunoassay (IMMULITE^®^ 2000 systems, Siemens Healthcare Diagnostics Products Ltd., Caernarfon, UK). IGF-I (nanograms per milliliter) was measured after employing excess IGF-II to saturate IGFBPs. The detection limit was 0.02 ng/mL. Interassay variance was 7.4 for IGF-I, and intraassay variance 5.6. Estradiol and testosterone concentrations were measured by chemiluminescent immunoassays using commercially available kits by Siemens Healthcare Diagnostics: Dimension Vista^®^ System Flex^®^ reagent cartridge LOCI^®^ Estradiol (AMR: 11–1500 pg/mL [40–5506 pmol/L]) and IMMULITE^®^ 2000 Total Testosterone (calibration Range: 20–1600 ng/dL (0.7–55 nmol/L)). Total cholesterol, triglycerides, high-density lipoprotein (HDL), and low-density lipoprotein (LDL) were measured using the Roche cobas^®^ c502 module. For the assessment of Vitamin D, a competitive immunoassay using the Roche cobas^®^ e601 module was performed. Intra- and interassay coefficients of variation were below 5% in all methods. Fasting blood glucose was measured via a UV test (hexokinase method with Roche cobas^®^ c502 module). Fasting insulin levels were determined with a sandwich immunoassay (ADVIA Centaur^®^ XPT immunoassay system, Siemens Healthineers). HbA1c was determined immunochemically through an antibody-based immunoassay (Roche cobas^®^ c502 module).

### 2.4. The Questionnaire

Parents and caregivers of all participants were asked to complete a questionnaire comprising questions of five thematic blocks. The questionnaire can be found in the Supplementary Material (S1). Firstly, responders were asked to describe the living situation, including parental occupation. The second block contained questions about the care and therapies received pre-, intra-, and post-lockdown. Questions of the third block covered the structure of everyday life during home confinement. Block number four contained questions about COVID-19 infections and other illnesses since the pre-lockdown assessment. The questions consisted of dichotomous, multiple-choice, scaling, and open questions. Lastly, parents or caregivers were asked to rate family life and their child’s behavior on a Likert scale (from 0: no behavioral problems to 9: severe behavioral problems). Improper answers were revoked and consequently excluded from the analysis. Answers were seen as improper when no or multiple options, instead of one, were chosen. No entire questionnaire was excluded. Questions applicable for all participants were answered by 80.0% to 99.3% of participants, five questions were answered by less than 80% (lowest response rate: 65.6%). 

### 2.5. Statistical Analysis

Statistical analysis was performed with IBM SPSS Statistics (versions 26 and 27). We calculated delta values (Δs), indicating differences between points of examination. To calculate Δ, the pre-lockdown data were subtracted from the corresponding post-lockdown data. No significant differences in Δ were found between female and male pairs, making a division into subgroups superfluous. Before testing, each parameter was evaluated for normal distribution. If the assumption of normality did not hold, non-parametric testing was performed. For measurements on ratio scales, paired t-tests or Wilcoxon signed-rank tests were used to compare values pre- and post-lockdown. For measurements on ordinal scales (Likert scales), sign tests were performed to compare pre-, intra-, and post-lockdown differences. For the assessment of associations between two variables, we used Pearson r and Spearman rho correlations. To further analyze determinants of pre- and post-lockdown differences, we performed stepwise multiple linear regression analyses in all participants older than two years of age. The Δ values were used as dependent variables. Independent variables included in the analysis were: sex, post-lockdown age, genetic subtype, GH dosage (mg/kg), number of siblings, support by other family members, hometown population, living situation (house/apartment), access to a garden, owning a dog, duration of break of childcare services (weeks), duration of break of physiotherapy (weeks), change in daily routine, presence of set mealtimes, presence of set activities, and behavior during the lockdown. Cohen f^2^ values were calculated to estimate effect sizes. Significance was defined as *p* ≤ 0.05. 

## 3. Results

### 3.1. Anthropometric Parameters and Body Composition

All the data are shown in Table 2. Weight-SDS_PWS_ and BMI-SDS_PWS_ decreased with no changes in fat mass in any age group. A statistically significant gain in LBM was found in all assessed age groups below the age of 18.

### 3.2. Hormonal Parameters, Lipoproteins, and Carbohydrate Metabolism

The data are shown in Table 3. IGFBP-3 concentrations rose significantly in all children, and IGF-I in all children below the age of 11. There were no significant changes in HbA1c concentrations in any group. Testosterone concentrations did not change significantly in boys aged 11–17-years, in those receiving testosterone replacement (pre-lockdown mean: 0.98 ng/mL vs. post-lockdown mean: 0.96 ng/mL, *p* = 0.893), nor in those not taking testosterone (pre-lockdown mean: 1.43 ng/mL vs. post-lockdown mean: 1.22 ng/mL, *p* = 0.570). Similarly, in the ≥18-year-olds taking testosterone (pre-lockdown mean: 4.35 ng/mL vs. post-lockdown mean: 4.43 ng/mL, *p* = 0.779) and those not taking testosterone (pre-lockdown mean: 2.98 ng/mL vs. post-lockdown mean: 2.61 ng/mL, *p* = 0.345), concentrations did not change significantly. In girls aged 11–17 years not receiving estradiol, concentrations increased significantly (pre-lockdown mean: 19.36 pmol/L vs. post-lockdown: 27.9 pmol/L, *p* = 0.032). Only two received estradiol replacement; therefore, no statistical analysis was performed. In the ≥18-year-old females, no statistical analysis was performed as concentrations were measured in three women. 

### 3.3. Growth Hormone Therapy and the Development of Weight/BMI/LBM

**Figure 1:**
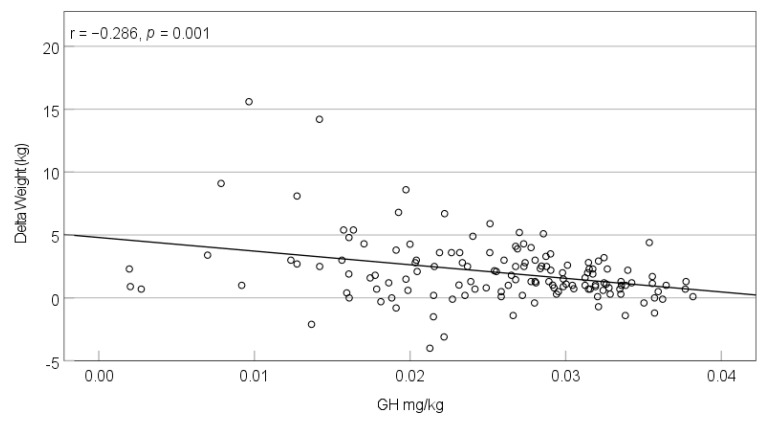
Correlation between the intra-lockdown GH dosage (mg/kg) and weight change (kg) during the lockdown period in March–April 2020 in individuals with PWS >2 years.

**Figure 2:**
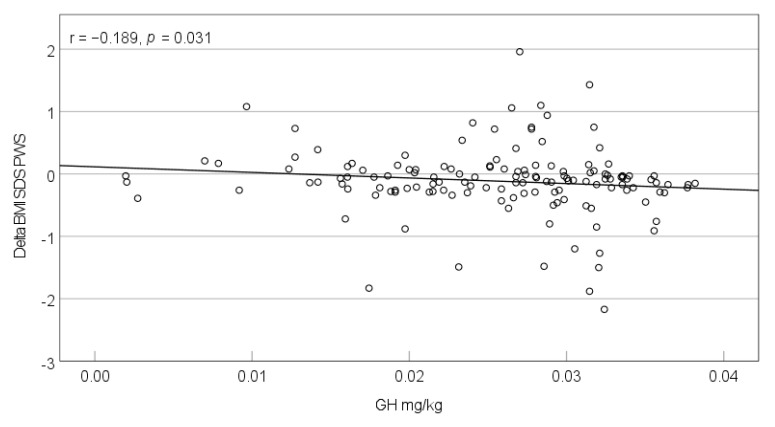
Correlation between the intra-lockdown GH dosage (mg/kg) and change in BMI-SDS_PWS_ during the lockdown period in March–April 2020 in individuals with PWS >2 years.

Most children (90.2%) and two adults (7%) received GH during the lockdown (Table 1). The dosage did not change significantly during the observational period (pre-lockdown mean: 0.0261 mg/kg vs. intra-lockdown mean: 0.0257 mg/kg, *p* = 0.225). The correlation between dosage change (mg/kg) and the change in IGF-I concentration did not reach statistical significance (*p* = 0.697). Assessing the entire cohort above the age of two, we found significant correlations between the GH intra-lockdown dosage (mg/kg) and the development of weight (kg) (r = −0.286, *p* = 0.001; Figure 1), weight-SDS_PWS_ (r = −0.183, *p* = 0.037), and BMI-SDS_PWS_ (r = −0.189, *p* = 0.031; Figure 2). The correlation between GH dosage and LBM development was non-significant (r = −0.107, *p* = 0.274). Calculated for the different age groups, correlations between GH dosage and weight-SDS_PWS_/BMI-SDS_PWS_ only remained significant in the 2–6-year-olds (weight-SDS_PWS_: r = −0.344, *p* = 0.010; BMI-SDS_PWS_: r = −0.365, *p* = 0.006). 

### 3.4. The Questionnaire

All participants completed the questionnaire. None of the individuals with PWS suffered from a COVID-19 infection. Forty-two children and adolescents (23.8%) contracted another illness (in 92.9%, an upper respiratory tract infection, gastroenteritis, or ear–nose–throat condition), five children (2.8%) had a scheduled surgery, and 22 (12.4%) received a new medication (in 95.5%, a non-steroidal anti-inflammatory drug, antibiotic, antipsychotic, or inhalative agent). One family lost a family member due to a COVID-19 infection. 

#### 3.4.1. Living Environment

Most families lived in a rural living environment defined as < 20,000 inhabitants (56.6%) and owned a house (74.1%) with a garden (73.1%). A total of 16% of children with PWS were a single child. About half of the families (52.7%) experienced a change in their daily routine during the lockdown. Most families reported keeping set mealtimes (95.4%) and activities (72.2%). Overall, 47.2% reported family life during the lockdown as strenuous, 33.1% as very strenuous. The highest percentage of parents not describing the family situation as strenuous was found in the <2-year-olds with 37.5%, followed by the 2–6-year-olds with 22.6%. The highest percentages of parents rating family life as very strenuous were seen in the 11–17-year-olds (50%) and 7–10-year-olds (36.1%). Regarding occupational changes, 67 fathers (42.4%) and 63 mothers (45.7%) reported a change caused by the pandemic. A total of 39 fathers (27%) and 36 mothers (29.5%) were sent to work from home or for short-time work during the lockdown. 

#### 3.4.2. Care and Therapies during COVID-19 Lockdown

Most families (88.7%) reported a discontinuation of childcare services. Care facilities were closed between 1 and 25 weeks, with a median duration of 12 weeks. Every fifth individual with PWS (21.2%) received a form of emergency care, which reached pre-lockdown capacity in 21.4%. Regarding the different age groups, a break in childcare services occurred most frequently in the 7–10-year-olds (100%), 11–17-year-olds (95.5%), and 2–6-year-olds (93.3%). In the young adults, about half (54.2%) experienced a closure of care facilities. As 9 out of 10 <2-year-olds had been cared for at home previous to the lockdown, only one family reported a discontinuation of childcare services. At the time of completing the questionnaire, 56.7% of all families reported a restart of care services, reaching the pre-lockdown extent in 48.1%. Therapy sessions were canceled in most cases (86%), of which 17.1% were offered support by their therapists via video conferencing sessions, training schedules, video instructions, or phone calls. 

#### 3.4.3. Behavior Changes during COVID-19 Lockdown

Median behavioral ratings for the lockdown period were “0” for children <2 years, “2” for 2–6-year-olds, “4” for 7–10-year-olds, “5” for adolescents aged 11–17 years, and “3” for young adults ≥18 years of age. Overall, 81 parents and caregivers (45.7%) described an increase in behavioral problems during the lockdown; an improvement was reported in 22 cases (12.4%). Comparing the behavior pre- and post-lockdown, 89 parents and caregivers did not experience a behavioral change (55.6%), 54 reported a persistent increase in behavioral problems (33.7%), and 17 a decrease (10.6%). Regarding the different age groups, parental behavior ratings significantly increased from pre- to intra-lockdown in all children and adolescents above the age of 2. Comparing intra- and post-lockdown behavior, parental ratings decreased significantly in children aged 2–6 years and young adults. In the groups of the 7–10- and 11–17-year-olds, no significant changes in parental ratings comparing intra- and post-lockdown behavior were found. Data of the different age groups are presented in Table 4. 

### 3.5. Multiple Regression Analysis

The multiple regression analysis did not show a strong association between one factor and post-lockdown outcomes. 

## 4. Discussion

The purpose of this study was the assessment of the impact of the lockdown in March and April 2020 on children, adolescents, and young adults with PWS. Among the most important findings of this study were the changes seen in anthropometric parameters, especially regarding the development of weight/BMI and lean mass. Previous studies have shown weight gain among healthy children during times of school closure due to loss of structured physical exercise and an increase in free time commonly used for sedentary activities [17,18,19]. Studies conducted during the COVID-19 pandemic found that children were less active and more sedentary; therefore, concern about weight gain among children has been expressed [20,21,22,23]. As weight gain is part of the natural history of PWS, we hypothesized that weight and BMI would increase in children, adolescents, and young adults with PWS. Interestingly, our study showed contrary results: both weight-SDS_PWS_ and BMI-SDS_PWS_ lowered in all age groups, reaching statistical significance in some. We observed a significant decrease in weight-SDS_PWS_ in children aged 7–10 years, BMI-SDS_PWS_ decreased significantly in the <2- and 7–10-year-olds. Similarly, in adults, weight and BMI decreased, however, non-significantly. Even though weight and BMI did not decrease in some age groups, maintaining a stable weight and BMI is considered the opposite of the natural history of PWS and can be seen as a favorable outcome [3,10].

Regarding body composition, we found a statistically significant increase in lean mass in all children and adolescents. An increase in LBM could have been caused by increased total body water, which was only assessed in the minority of participants. However, none of the participants was reported to have cardiac failure, and only one suffered from beginning renal failure, making a gain in muscle mass more likely than water retention. 

There are various factors, which might explain the positive development of a stable weight/BMI and even improved body composition. First, we observed an increase in serum IGF-I and IGFBP-3 concentrations. Individuals with PWS show symptoms of GH deficiency, such as low IGF-I concentrations and abnormal body composition. Muscle mass is reduced, and fat mass increased, even in individuals with PWS with a normal BMI [7,10]. Therapy with GH increases IGF-I and IGFBP-3 concentrations and has been shown to have various benefits, including improvement of body composition and BMI [7,10]. In our cohort, 77.7% of all and 90.2% of individuals below the age of 18 received GH therapy during the lockdown. In most participants, the intra-lockdown GH dosage was determined at the pre-lockdown assessment in the clinic. When families were unable to attend the clinic, appointments were held over the phone. In these cases, GH dosages were adjusted based on laboratory tests performed by family physicians. An increase in prescribed GH dosage over the lockdown period could have explained the increased IGF-I and IGFBP-3 concentrations. However, as there was no difference in dosage adjusted to body weight received before and during the lockdown, and the correlation between changes in GH dosage and IGF-I concentrations was non-significant, a plausible alternative explanation for the increased concentrations might be an increase in therapy adherence. We did not directly assess therapy adherence, but it has previously been described to be suboptimal in pediatric populations [24]. An increase in GH therapy adherence during the COVID-19 pandemic was reported by Giavoli et al., indicating that home confinement influenced the adherence rate positively [25]. We therefore speculate that the individuals with PWS received a more continuous GH administration due to more flexible family routines and fewer travels during the lockdown. As mentioned, GH therapy is associated with improvements in body composition, including improvements in lean mass. However, we could not find a significant correlation between the dosage of intra-lockdown GH therapy and the observed positive change in LBM. This might be due to the little variation in GH dosage for the group. Only a small percentage of the population did not receive GH treatment; therefore, a comparison of the change in LBM during the lockdown between the non-treatment group versus the treatment group was not possible. Regarding the effect of GH on total body weight and BMI of the entire cohort, we found significant correlations between the intra-lockdown GH dosage and change of weight-SDS_PWS_ and BMI-SDS_PWS_. Higher dosages were associated with a reduction in weight and BMI. Calculated for the different age groups, these associations remained significant in children aged 2–6 years. Reasons for the statistically significant results in this age group might be the natural history of PWS, with a switch from failure to thrive to weight gain in this age range, higher therapy adherence in children than adolescents, or a larger sample size compared to the other groups [25]. 

Another common hormone deficiency in PWS is hypogonadism, and many adolescents and young adults receive sex steroid replacement therapy [7,26]. Apart from induction and progression of puberty, the benefits of sex steroid replacement, specifically testosterone replacement, include improvements in muscle mass and body composition. Individuals of two age groups received sex steroids: adolescents between 11 and 17 years of age and young adults ≥18 years. However, in the 11–17-year-olds, only a minority received testosterone, as parents are commonly hesitant towards testosterone treatment, fearing an increase in behavioral problems. Moreover, in all adolescent boys, serum testosterone concentrations did not differ significantly pre- and post-lockdown. In the young adults, a majority took sex steroids, but no significant changes in weight and body composition were found. Therefore, we assume that sex steroid replacement therapy did not significantly impact the development of weight and lean mass in either age group. 

As a second factor possibly influencing weight and body composition, we identified physical activity. Physical activity is a recommended treatment component, as it improves physical capacity and aids obesity management [11,27]. Regarding body composition, the impact of physical activity on LBM in individuals with PWS is ambiguous [28,29,30]. We did not directly assess the level of physical activity in our participants, but families reported keeping routines during the lockdown, and elevated vitamin D levels in the post-lockdown assessment indicate that families spent time outdoors. Keeping routines and being physically active, when possible outside, have been recommended to maintain healthy movement behaviors during the COVID-19 pandemic [31,32]. Supportive of the theory that enhanced physical activity impacted weight management, our regression analysis showed negative associations between set activities and changes of BMI-SDS_PWS_ and weight-SDS_PWS_, meaning that the presence of set activities caused lower BMI-SDS_PWS_ and weight-SDS_PWS_. However, these associations only had a weak and weak-moderate effect, respectively. 

A third possible factor was changes in eating behavior. As most families spent their days together at home, parents had greater control over the food consumed by their children, and almost all families (95.4%) reported having structured mealtimes, a recommended obesity management strategy in PWS [11]. Continuous caregiver supervision was described to have created a favorable environment for adults with PWS by Mosbah et al., causing weight loss in 49% of French adults with PWS during the lockdown period [33]. Regarding metabolic changes, we observed an increase in HDL and a reduced triglyceride/HDL ratio in children. Together with the anthropometric results, this may reflect a more balanced diet and regular physical activity over the lockdown period [34,35]. However, improved lipid profiles have also been linked to GH therapy [36,37]. The slightly worsened blood glucose and insulin concentrations observed in some age groups might have been caused by the diabetogenic effects of the suspected GH increase [38]. 

We, therefore, suggest that the favorable development seen in weight/BMI and body composition results from an interplay of the suspected enhanced GH therapy and parental commitment. As discussed above, GH therapy improves body composition and physical capacity. However, the changes in LBM in our population cannot solely be explained by GH treatment. Parental encouragement possibly led to increased physical activity and greater control over the food consumed, both recommended in the management of genetic obesity during the pandemic, also impacting weight/BMI and LBM [39]. Our multiple regression analyses further support the theory of a multifactorial process. To identify variables possibly impacting post-lockdown outcomes, we performed multiple regression analyses, including variables possibly affecting the development of weight and body composition. We differentiated between genetic subtypes, sex, post-lockdown age, and GH dosage, and included information given in the questionnaire, such as variables impacting physical activity (access to a garden, owning a dog, presence of set activities, living situation, and rural vs. urban neighborhood), eating behavior (presence of set mealtimes), or care and therapy received during the lockdown period. None of these variables showed a strong association with post-lockdown outcomes, showing that no single factor alone can be attributed to the observed changes.

We believe that these results might be reassuring to parents and caregivers of individuals with PWS, showing that the break of childcare services and therapies did not worsen weight and body composition. However, the results greatly depended on parental and caregiver commitment as external support was scarce during the lockdown, putting a significant burden on the families. 

PWS negatively impacts the quality of life of individuals with PWS and their parents even under non-pandemic circumstances [40]. During the pandemic, Wieting et al. reported an increase in temper outbursts in 51.7% and increased irritability in 55% of individuals with PWS [41]. In our population, 45.7% of parents and caregivers reported more intense behavioral problems during the lockdown period, and family life was described as strenuous/very strenuous in 80.3%. Regarding the different age groups, significant increases in behavioral problems were reported in all children and adolescents above the age of 2. In young adults, a non-significant trend towards an increase in behavioral problems was noted. In children aged 2–6 years and young adults, parental ratings decreased significantly post-lockdown, but not in children and adolescents aged 7–10 and 11–17 years. Instead, significant differences between pre- and post-lockdown behavior were reported in these two groups, showing that the intensified behavioral problems persisted into the post-lockdown period. Moreover, median behavioral ratings were highest in these two age groups; therefore, it is not surprising that the percentages of parents/caregivers describing family life as very strenuous were highest in these groups. Mental and behavioral problems, as well as caregiver burden, have been described to be highest in adolescents, even under non-pandemic circumstances [42]. Additionally, as the closure rates of care services in these groups were highest, it is likely that children and adolescents in the 7–10- and 11–17-year-old groups and their families suffered most during the COVID-19 lockdown. 

Overall, with more intense behavioral problems persisting after the first lockdown in March–April 2020 in one-third of all participants, the families’ burden continued into the post-lockdown period and was potentially further worsened by following lockdowns during the ongoing pandemic. 

### Strengths and Limitations

An increasing amount of research is being conducted assessing the mental and physical consequences of the COVID-19 pandemic on children. To the best of our knowledge, this is the first study evaluating the impact of the lockdown on weight and body composition of children, adolescents, and young adults with PWS. A strength of this single-center study is the evaluation of a large study cohort. In total, 231 individuals with PWS visited the clinic throughout 2020, of which we included 77.9% in our study, making the cohort representative for the population seen in our clinic. One important limitation is the two examinations not occurring right before and after the lockdown. Moreover, we did not use a standardized follow-up, nor questionnaire, and many study results rely on retrospective self-reporting. Furthermore, we did not assess therapy adherence. Due to missing memory functions in GH preparations licensed for use in PWS in Germany, an objective assessment of therapy adherence was not possible. Next, a pubertal growth spurt in the 11–17-year-olds could have impacted the development of height, weight, and BMI. It must also be noted that more than 70% of our population lived in a house with access to a garden. Therefore, our results might not be transferrable to populations living under less privileged conditions. Moreover, total body water was only assessed in the minority of participants. We can only assume that the increase in LBM was caused by an increase in muscle mass rather than water retention. In future research, complete body composition analyses should be performed in all participants. Further, due to the low percentage of individuals not receiving GH therapy, no statistical analysis investigating these children and adolescents separately was performed. Therefore, we could not identify the separate impacts of GH therapy and lifestyle variables on post-lockdown outcomes.

## 5. Conclusions

This study assessed the impact of the first COVID-19 lockdown on children, adolescents, and young adults with PWS living in Germany, showing a favorable development of weight/BMI and LBM. We suggest that these outcomes were caused by an interplay of enhanced GH administration and continuous parental supervision. Caring for their children with PWS without or only minimal support put a significant burden on the families. Enhanced behavioral problems were observed in almost half of the individuals with PWS, which persisted in one-third into the post-lockdown period. Our results highlight the beneficial effects of a multifactorial therapy comprising GH treatment, physical activity, and diet control. However, the results also stress the importance of multidisciplinary care to mitigate the burden on individuals with PWS and their families.

## Figures and Tables

**Table 1:** Characteristics of children and adolescents with PWS during the lockdown in March–April 2020.

	<2 Years	2–6 Years	7–10 Years	11–17 Years	≥18 Years
Sample size (n)	10	59	38	46	27
Mean age in years (range)	1.38 (0.94–1.96)	4.07 (2.02–6.94)	8.92 (7.05–10.95)	14.33 (11.26–17.99)	23.38 (18.49–33.55)
Females (%)	10 (100%)	31 (52.5%)	19 (50%)	23 (50%)	12 (44.4%)
Median interval between assessments (months)	5	6	6.5	7	9
n GH therapy pre-lockdown (%)	5 (50%)	55 (93.2%)	37 (97.4%)	34 (73.9%)	2 (7.4%)
n GH therapy intra-lockdown (%)	10 (100%)	56 (94.9%)	38 (100%)	34 (73.9%)	2 (7.4%)

Abbreviation: growth hormone (GH).

**Table 2:** Anthropometric parameters before and after the lockdown in March–April 2020. *p*-values > 0.05 depicted as “NS”.

		<2 Years			2–6 Years			7–10 Years			11–17 Years			≥18 Years		
		Mean	SD	*p*	Mean	SD	*p*	Mean	SD	*p*	Mean	SD	*p*	Mean	SD	*p*
Height (m)	*Pre*	**0.67**	0.08	**0.005**	**0.96**	0.12	**<0.001**	**1.29**	0.10	**<0.001**	**1.55**	0.12	**<0.001**	1.67	0.12	NS
	*Post*	**0.77**	0.06		**1.00**	0.12		**1.33**	0.10		**1.57**	0.11		1.67	0.11	
Height SDS_PWS_	*Pre*	**0.15**	0.69	**0.028**	0.60	0.96	NS	0.61	0.60	NS	**1.19**	0.93	**<0.001**			
	*Post*	**0.72**	0.93		0.57	1.01		0.66	0.61		**1.28**	0.91				
Weight (kg)	*Pre*	**6.93**	1.17	**0.005**	**14.52**	5.15	**<0.001**	**30.93**	9.09	**<0.001**	**54.92**	18.00	**<0.001**	69.07	9.75	NS
	*Post*	**9.04**	1.52		**16.32**	5.91		**33.17**	9.99		**57.52**	19.72		67.87	11.92	
Weight SDS_PWS_	*Pre*	0.12	0.70	NS	−0.73	0.79	NS	**−0.78**	0.73	**0.044**	−0.71	0.90	NS			
	*Post*	−0.15	0.83		−0.81	0.80		**−0.82**	0.77		−0.75	0.94				
BMI (kg/m^2^)	*Pre*	15.30	1.31	NS	15.51	2.09	NS	18.35	3.76	NS	22.38	5.23	NS	24.75	3.07	NS
	*Post*	15.15	1.66		15.88	2.55		18.45	3.68		22.77	5.99		24.20	3.30	
BMI SDS_PWS_	*Pre*	**−0.30**	1.25	**0.037**	−1.15	0.73	NS	**−1.24**	0.67	**0.007**	−1.36	0.65	NS			
	*Post*	**−1.08**	1.29		−1.21	0.77		**−1.32**	0.65		−1.39	0.71				
LBM (kg)	*Pre*				**13.72**	3.17	**<0.001**	**21.97**	4.58	**<0.001**	**35.58**	8.89	**<0.001**	44.94	7.93	NS
	*Post*				**15.11**	3.49		**23.43**	5.41		**37.24**	9.99		43.26	9.56	
Fat Mass (%)	*Pre*				17.43	7.51	NS	26.62	7.89	NS	32.01	8.68	NS	36.14	7.40	NS
	*Post*				17.02	9.07		26.75	7.41		32.27	8.73		35.21	6.62	

Abbreviations: standard deviation scores for children with Prader–Willi syndrome (SDS_PWS_), body mass index (BMI), lean body mass (LBM).

**Table 3:** IGF-I and IGFBP-3, lipoproteins, and carbohydrate metabolism before and after the lockdown in March–April 2020. *p*-values > 0.05 depicted as “NS”.

		<2 Years			2–6 Years			7–10 Years			11–17 Years			≥18 Years		
		Mean	SD	*p*	Mean	SD	*p*	Mean	SD	*p*	Mean	SD	*p*	Mean	SD	*p*
IGF-1 (µg/L)	*Pre*	**46.97**	29.42	**0.008**	**115.17**	58.46	**<0.001**	**233.25**	74.29	**0.022**	307.19	109.17	NS	146.63	46.46	NS
	*Post*	**81.73**	41.63		**147.61**	59.31		**264.11**	108.65		319.13	118.17		148.63	71.97	
IGFBP-3 (mg/L)	*Pre*	**2.56**	0.85	**0.008**	**4.09**	1.11	**<0.001**	**5.51**	1.00	**<0.001**	**6.70**	1.41	**0.001**	5.65	1.27	NS
	*Post*	**4.13**	1.18		**5.01**	1.16		**9.01**	16.39		**7.41**	1.72		5.88	1.19	
Total cholesterol (mg/dL)	*Pre*	139.22	31.91	NS	163.56	28.79	NS	157.92	27.14	NS	155.52	29.45	NS	158.50	37.06	NS
	*Post*	150.22	17.35		167.81	27.55		164.22	21.15		156.82	27.03		159.42	37.34	
Triglycerides (mg/dL)	*Pre*	73.56	17.07	NS	**76.83**	30.09	**0.047**	62.39	20.74	NS	**77.57**	37.99	**0.050**	88.19	54.66	NS
	*Post*	67.89	20.76		**69.97**	20.52		66.42	25.62		**95.45**	78.58		84.85	44.61	
HDL (mg/dL)	*Pre*	39.81	9.02	NS	**49.82**	11.00	**<0.001**	**58.23**	15.12	**0.008**	53.14	11.11	NS	52.05	14.24	NS
	*Post*	45.60	8.23		**55.38**	9.62		**63.19**	11.93		53.07	11.77		52.45	12.16	
LDL (mg/dL)	*Pre*	84.78	25.92	NS	98.80	26.99	NS	87.25	20.54	NS	88.00	23.97	NS	90.96	31.75	NS
	*Post*	91.11	14.13		98.58	25.90		87.72	19.86		87.05	23.75		90.92	32.47	
Fasting glucose (mg/dL)	*Pre*	82.33	3.24	NS	81.44	9.48	NS	88.00	7.43	NS	90.44	7.56	NS	88.81	7.12	NS
	*Post*	80.78	8.71		85.63	12.44		89.31	8.43		91.76	7.90		96.40	32.63	
OGTT 30 min (mg/dL)	*Pre*	127.60	17.57	NS	145.91	28.75	NS	**152.47**	31.19	**0.035**	155.14	23.70	NS	149.21	30.37	NS
	*Post*	134.20	33.25		149.64	27.17		**165.14**	28.53		158.11	26.80		158.63	34.50	
OGTT 60 min (mg/dL)	*Pre*	112.60	28.84	NS	126.77	26.58	NS	133.61	19.87	NS	142.66	30.74	NS	**139.92**	42.93	**0.006**
	*Post*	124.60	43.33		129.88	31.62		137.11	32.15		148.82	36.65		**157.17**	41.88	
OGTT 120 min (mg/dL)	*Pre*	111.60	32.76	NS	**105.61**	17.67	**0.026**	**110.47**	19.43	**0.006**	114.76	22.14	NS	111.76	20.44	NS
	*Post*	109.60	21.42		**111.50**	19.35		**121.31**	20.99		123.55	31.46		121.67	32.13	
Insulin (pU/dL)	*Pre*	1.89	1.08	NS	4.17	3.56	NS	**6.59**	2.71	**0.042**	11.61	5.59	NS	**6.99**	3.39	**0.003**
	*Post*	2.42	1.70		4.24	3.05		**7.79**	3.79		15.34	19.21		**5.12**	1.76	
Vitamin D (nmol/L)	*Pre*	92.75	66.40	NS	**65.43**	23.91	**<0.001**	74.44	48.48	NS	**66.59**	29.19	**0.004**	**75.50**	23.99	**0.001**
	*Post*	88.70	50.91		**80.04**	22.33		75.44	22.55		**76.70**	26.45		**97.79**	21.93	

Abbreviations: insulin-like growth factor 1 (IGF-I), insulin-like growth factor-binding protein 3 (IGFPB-3), high-density lipoprotein (HDL), low-density lipoprotein (LDL), oral glucose tolerance test (OGTT).

**Table 4:** Behavioral problems reported by parents.

	<2 Years				2–6 Years				7–10 Years				11–17 Years				≥18 Years			
	Less	Equal	More	*p*	Less	Equal	More	*p*	Less	Equal	More	*p*	Less	Equal	More	*p*	Less	Equal	More	*p*
Intra-pre-lockdown	2	6	0	0.500	3	35	20	**0.000**	6	12	20	**0.011**	6	13	27	**0.000**	5	8	14	0.064
Post-intra-lockdown	0	7	0	1.000	12	36	3	**0.035**	13	13	8	0.383	15	21	8	0.210	12	10	2	**0.013**
Post-pre-lockdown	2	5	0	0.500	3	41	7	0.344	2	16	16	**0.001**	6	18	20	**0.011**	4	9	11	0.118

Bahvioral problems reported by parents for each participant with PWS comparing intra- vs. pre-, post- vs. intra-, and post- vs. pre-lockdown behavior. Behavioral problems were rated on a 0 to 9 Likert scale, “0” meaning no behavioral problems, “9” severe behavioral problems.

## Data Availability

The datasets generated during this study are not publicly available in order to protect the privacy of the patients who participated in this study.

## Supplementary Materials

The following are available online at https://www.mdpi.com/article/10.3390/jcm10204746/s1, S1: The Questionnaire.
