# Supplementary material for: Effects of COVID-19 Lockdown on Weight, Body Composition, and Behavior of Children, Adolescents, and Young Adults with Prader–Willi Syndrome
Source: J Clin Med. 2021 Oct 16;10(20):4746. doi: 10.3390/jcm10204746 (PMC8541437; doi:10.3390/jcm10204746)
Supplement: Supplementary file 1 [file jcm-10-04746-s001.zip › jcm-1405480-supplementary.pdf]

Children and Adolescents with PWS during the COVID-19 Pandemic

Family situation since the Lockdown
(Please tick appropriate answer)

Father: Occupation____

Occupational situation since the Lockdown

[ ] unchanged [ ] changed

- [ ] unchanged at work
- [ ] self-employed
- [ ] shiftwork
- [ ] working from home
- [ ] short-time work
- [ ] other

Mother: Occupation ____

Occupational situation since the Lockdown

[ ] unchanged [ ] changed

- [ ] unchanged at work
- [ ] self-employed
- [ ] shiftwork
- [ ] working from home
- [ ] short-time work
- [ ] other

**Siblings:**	Date of birth:	Educational level/occupation:

Did your family receive support by other family members?

[ ] No [ ] Yes

Whom :____

Hometown: Population:________________________________

Childcare during the Lockdown

Childcare before COVID-19: [ ] At home [ ] Nursery [ ] School

Grade ________________________

Mainstem institution [ ] Institution with inclusion [ ]

Other: ____

Break in childcare services: [ ] Yes [ ] No

[ ] **Emergency childcare**; extend of emergency childcare:

____

Duration; from: ___________________________ to: _________________________________

Therapies

	Before COVID-19	Break from:	to:
Physiotherapy			
Occupational therapy			
Speech therapy			
Early intervention			
Therapeutic riding			

Support received from therapists,
e.g., through video instructions, therapy plans, etc.

[ ] No [ ] Yes

Which form of support was offered by the therapists:

____

____

____

____

____

Family Life during the COVID-19 Lockdown

Change in routines	[ ] No	[ ] Yes
Set mealtimes	[ ] No	[ ] Yes
Breakfast time	________________________________ o'clock	
Set activities	[ ] No	[ ] Yes

Which set activites:

____

____

____

Medical situation

Has your child or another family member been infected with COVID-19?

[ ] No [ ] Yes

Who:____

Severity/course of infection:

____

Has your child been sick since the last appointment?

[ ] No [ ] Yes

Disease:____

Did your child have to take any new medications?

[ ] No [ ] Yes

Which medications:____

How did your child with PWS deal with the new situation?

Behavioral problems (0 = no behavioral problems 9 = severe behavioral problems)

Before the Lockdown

0	1	2	3	4	5	6	7	8	9

During the Lockdown

0	1	2	3	4	5	6	7	8	9

After the Lockdown

0	1	2	3	4	5	6	7	8	9

How would you rate your family situation during the Lockdown?

Unchanged compared to before the Lockdown [ ] No [ ] Yes

Strenous [ ] No [ ] Yes

Very strenuous [ ] No [ ] Yes

Our family moved closer together [ ] No [ ] Yes

Our family enjoyed the lockdown [ ] No [ ] Yes

Room for Comments

e.g., what did your family do differently during the Lockdown?

____

____

____

____

____

What kind of support would you wish to receive during a second lockdown?

____

____

____

____

____
